# From genes to diagnosis: The impact of UNC5B and DOK5 in intracranial aneurysm detection and pathogenesis

**DOI:** 10.1371/journal.pone.0340496

**Published:** 2026-03-13

**Authors:** Zekun Ma, Pengfei Wu, Alimasi Abulizi, Wenbo Yang, Aierpati Maimaiti, Paziliya Akram, Zengliang Wang

**Affiliations:** Department of Neurosurgery, The First Affiliated Hospital of Xinjiang Medical University, Urumqi, China; Indiana University School of Medicine, UNITED STATES OF AMERICA

## Abstract

**Objective:**

Intracranial aneurysms exhibit a notable prevalence within the general population, characterized by an incidence rate ranging from 1% to 2% and an annual rupture rate of approximately 16.4 per 100,000 individuals.Genes that are diagnostic and therapeutic are being investigated in this study linked to intracranial aneurysms by integrating machine learning, immune infiltration analysis, and single-gene sequencing.

**Methods:**

Differentially expressed genes (DEGs) were identified based on microarray data from the Gene Expression Omnibus (GEO) database between individuals with intracranial aneurysms and healthy controls.The DEGs were functionally analyzed using Gene Ontology (GO),Disease Ontology (DO) and Kyoto Encyclopedia of Genes and Genomes (KEGG) pathways.Diagnostic biomarkers were identified and validated using machine learning algorithms and confirmed with external validation datasets.Subsequently, circulating biomarkers were assessed, and immune cell infiltration analysis along with single-cell sequencing were employed to elucidate the functional roles of the selected diagnostic biomarkers.

**Results:**

It was found that intracranial aneurysm patients and healthy controls shared 13,101 DEGs, with 1,108 genes upregulated and 969 downregulated.Aneurysms containing intracranial aneurysms showed significant immunoresponse-related enrichment in GO,DO,and KEGG pathway analyses.Technologies based on machine learning, such as LASSO, Random Forest, and SVM-RFE(Support Vector Machine-Recursive Feature Elimination), identified UNC5B and DOK5 as potential diagnostic biomarkers with high efficacy. Immune cell infiltration analysis indicated an elevated presence of various immune cells in intracranial aneurysms, particularly M1 macrophages.The UNC5B gene expression in fibroblasts is linked to intracranial aneurysm pathogenesis.

**Conclusion:**

In conclusion, the UNC5B and DOK5 genes emerge as potential diagnostic biomarkers for intracranial aneurysms.There is a positive correlation between UNC5B expression and M1 macrophages and it is primarily found in fibroblasts, suggesting that increased M1 macrophages and UNC5B expression in fibroblasts may contribute to intracranial aneurysm pathogenesis.

## 1. Introduction

Intracranial aneurysms represent one of the most prevalent cerebrovascular disorders, most frequently, saccular (berry) aneurysms cause localized dilation of cerebral arteries [1]. It is estimated that about 2% of the population has intracranial aneurysms.However, prospective angiographic studies have reported prevalence rates as high as 6.0% [2]. These aneurysms predominantly manifest at arterial bifurcations, Willis’ circle accounts for 85% of the cases.Most intracranial aneurysms affect the anterior circulation(approximately 90%), specifically the internal carotid artery.Aneurysms located in the anterior communicating artery make up 35% of all aneurysms, it is composed of 30% internal carotid arteries and 22% middle cerebral arteries.A majority of aneurysms in the posterior circulation occur in the basilar artery, which accounts for 5% of cases. [3]. Approximately 30% of patients exhibit multiple aneurysms [2]. It is prevalent for patients to present with sudden, severe headaches attributed to a ruptured aneurysm, causing subarachnoid hemorrhage. Patients usually present with a sudden, severe headache. Nausea, vomiting, lethargy, confusion, and loss of consciousness are common symptoms, visual disturbances, and meningismus. [4]. Research findings indicate that 17% of patients experience immediate mortality [5], while an additional 25% die during hospital treatment. [6]. Outcomes can be prognosticated based on three primary factors: the age of the patient, his or her clinical condition upon admission according to the Hunt and Hess grading scale, and any occurrence of vasospasm. [7]. The incidence of rebleeding is most pronounced within the initial 24 hours following rupture, with an estimated rate of approximately 15% [8]. Subsequently, around 20% rebleeding rate is observed in aneurysms events occur within the initial two weeks post-rupture, 30% within the first month, and 50% within the first six months.Recurrent subarachnoid hemorrhage has a mortality rate of up to 50%,with the risk of rebleeding decreasing over time to about 3% annually. The repercussions of aneurysm rupture are profound, with one-third of survivors requiring ongoing caregiver support and only 20% preserving their pre-rupture quality of life [9].

Clinicians’ therapeutic decisions have been greatly influenced by advances in treatment modalities for intracranial aneurysms (IAs).Endovascular approaches such as coiling, balloon- or stent-assisted coiling, endovascular flow diversion, and flow disruption may be used to treat IAs surgically.There are instances when a singular approach might not be sufficient to resolve an issue in its entirety, necessitating the integration of both microsurgical and endovascular techniques, particularly in the management of complex aneurysms.Treatment strategies must take into account various factors, including age, patient preferences, aneurysm location, shape, and size, morphology,medical history, the operator’s experience as well as comorbidities [10]. Consequently, a comprehensive investigation into the pathogenesis of intracranial aneurysms is essential for identifying potential biomarkers.These biomarkers would improve intracranial aneurysm diagnosis and facilitate the creation of new molecular therapies.Implementing these strategies enables early-stage targeted drug interventions for aneurysm progression, thereby reducing patient mortality.

In intracranial aneurysms, pathological changes are mainly caused by he breakdown of extracellular matrix, vascular smooth muscle cell dysfunction, and loss of internal elastic lamina.A variety of vascular risk factors as well as genetic predispositions may cause intracranial aneurysms.Genetic susceptibility increases the risk of mechanical damage at vascular bifurcations, possibly due to congenital structural defects or localized metabolic abnormalities influenced by blood flow dynamics.The endothelial layer initially sustains damage, this causes a cascade of changes within the vascular wall, including lipid deposition and hemodynamic changes.As inflammation cells infiltrate the affected area, they release mediators, causing smooth muscle cells to undergo a phenotypic change from contractile to secretory.Blood flow and lipid deposition exacerbate microstructural changes in the vascular wall, amplifying local inflammation. Apoptosis is induced in smooth muscle and endothelial cells, reducing vascular wall strength and leading to aneurysm formation.The local metabolic environment, ongoing changes in hemodynamics, and inflammatory responsest further weaken the vessel wall’s structural integrity, promoting aneurysm progression until rupture [11].

The metabolism of lipids, including triglycerides, cholesterol, and fatty acids, involves the synthesis and breakdown of these substances.In addition to transporting lipids between the liver and peripheral tissues, special lipoproteins transport lipids from the intestines to the liver.Dyslipidemia is defined by aberrant levels of lipids or lipoproteins [12]. Prior research has identified specific cytokines as potential biomarkers linked to the progression of intracranial aneurysms.These biomarkers are linked to various pathological processes, including smooth muscle cell phenotypic transformation, endothelial dysfunction, walls of the arteries are infiltrated and accumulated with inflammation cells, and the expression and release of chemokines (e.g., IL-8,MCP-1), extracellular matrix remodeling proteases (e.g., MMP-9, MMP-2), pro-inflammatory cytokines (e.g., TNF-α, IL-1β), and cell adhesion molecules (e.g., ICAM-1, VCAM-1). Intracranial aneurysms are caused by a combination of factors. [13,14]. NF-B transcriptional activity regulates matrix metalloproteinases and pro-inflammatory markers [14]. There is evidence that activated NF-B increases matrix metalloproteinases and inflammatory markers expression, while its inhibition reduces their expression [15–17]. Bioinformatics analysis has recently become crucial for understanding disease mechanisms, aided by various sequencing technologies that reveal transcriptome-level gene expression profiles [18].

In machine learning, predictive models are created based on data or discerning patterns within datasets.This discipline inherently strives to replicate or approximate human pattern recognition abilities through computational means [19]. Unsupervised learning and supervised learning are both types of machine learning. In the medical field, supervised learning is most commonly used, involving the computer identifying patterns from labeled datasets with predefined target labels.These labels furnish feedback to the computational algorithm, thereby enhancing its predictive accuracy.A variety of supervised learning models, including logistic regression and support vector machines,linear regression, and tree-based models like XGBoost and random forests, are relevant to numerous medical tasks.Tree-based algorithms are frequently cited in literature reviews and consistently demonstrate robust performance in medical applications [20]. These models employ decision trees, the model development phase involves the development of intricate decision points based on training data.In contrast, an unsupervised learning algorithm involves presenting a model with an unlabeled dataset so that it can identify and describe relationships in the data by looking for patterns and trends within the dataset.The discovery of unknown patterns relies heavily on unsupervised learning [21,22]. Among the models used in unsupervised learning arethe principal component, the k-nearest neighbors, and the k-means clustering analysis.

This study utilized bioinformatics to analyze gene expression profiles from normal and intracranial aneurysm samples using gene chips and single-cell sequencing data.Machine learning techniques identified two potential diagnostic biomarker genes.Immune infiltration analysis identified potential mechanisms and therapeutic targets for intracranial aneurysm progression and pathogenesis.

## 2. Research methods and data

### 2.1. Data sources and preprocessing

**Fig 1:**
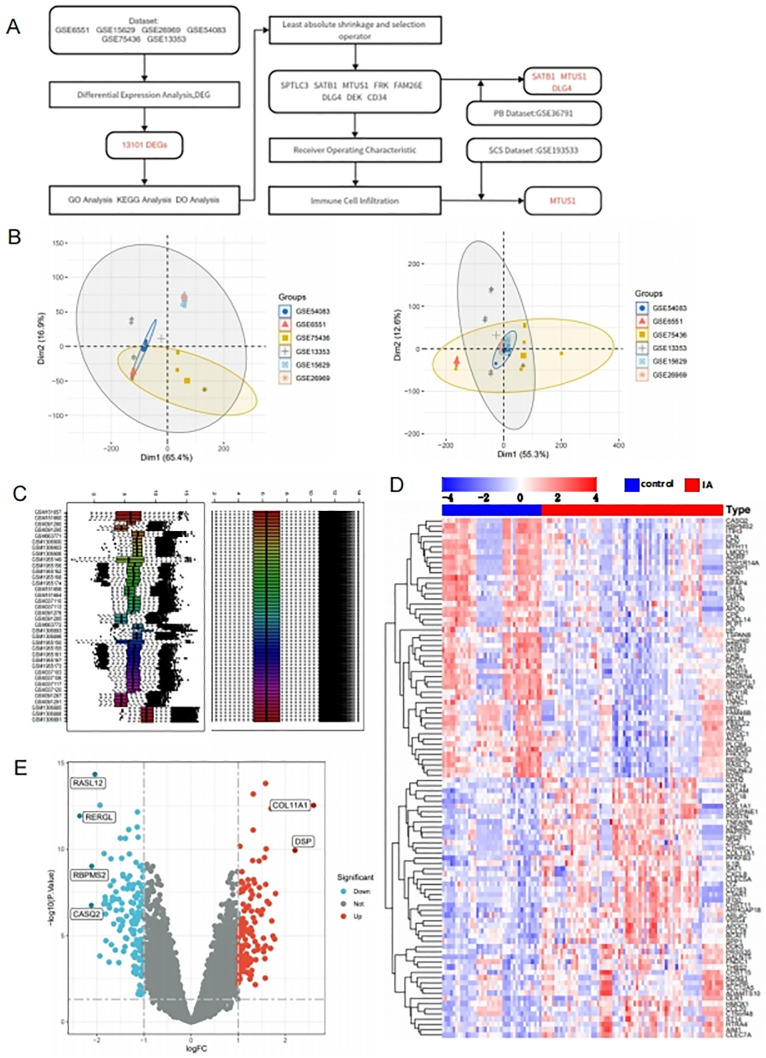
Data Collection and Analysis of DEGs (A: Analytical workflow of the study; B: PCA plot of gene expression datasets, with different colors representing samples from different datasets; C: Box plot of gene expression datasets; D: Heatmap and clustering analysis of DEGs between the control and IA groups, with red indicating high expression levels and blue indicating low expression levels; E: Volcano plot of DEGs between the control and IA groups, with red indicating upregulated genes, blue indicating downregulated genes, and black indicating genes with no differential expression. Genes with logFC > 2 are highlighted.).

Gene Expression Omnibus (GEO) (https://www.ncbi.nlm.nih.gov/geo/) data were used to obtain GSE6551,GSE15629,GSE26969,GSE54083,GSE75436,GSE13353,GSE193533,andGSE36791.Of Which GSE6551 comprised 5 intracranial aneurysm samples and 5 normal intracranial artery samples.GSE15629 contained 14 intracranial aneurysm samples, subdivided into 8 ruptured intracranial aneurysm samples and 6 unruptured Iintracranial aneurysm samples, along with 5 normal samples.GSE26969 contained 3 IA samples and 3 normal samples.GSE54083 comprised 13 IA samples, including 8 RIA and 5 UIA, also 10 normal samples.GSE75436 contained 15 IA samples besides 15 normal samples.GSE13353 consisted of 14 IA samples, with 11 RIA and 8 UIA.GSE193533, a mouse single-cell sequencing dataset, and GSE36791, containing peripheral blood samples, underwent background correction and batch effect mitigation making use of the “ComBat” function from the “sva” R package (version 3.50.0).Subsequently, the data were merged and normalized.(Fig 1A, B).

### 2.2. Gene expression analysis of differentially expressed genes

**Fig 2:**
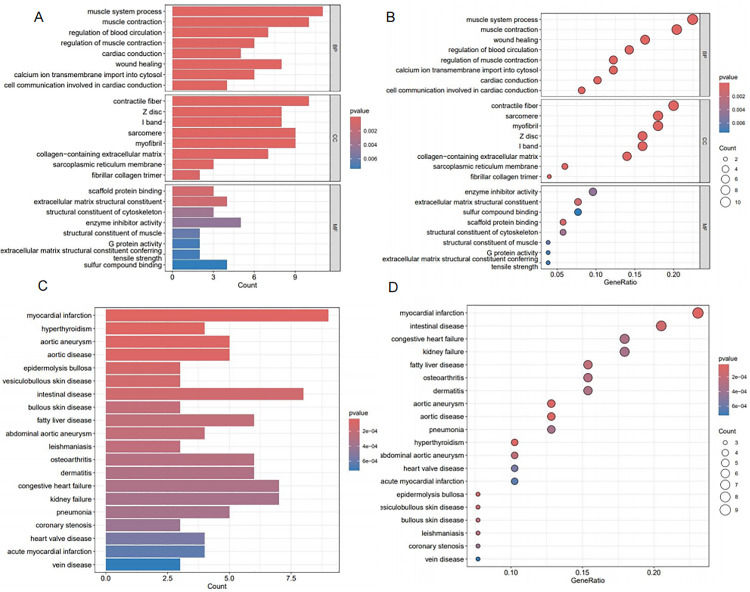
Functional and Disease Enrichment Analysis of DEGs (A-B: Bar chart (A) and bubble chart (B) of GO enrichment analysis for DEGs in the datasets; C-D: Bar chart (C) and bubble chart (D) of DO enrichment analysis for DEGs in the datasets).

By using the R package “limma” (version 3.58.1) we identified DEGs with a threshold of P < 0.05 and |log2FoldChange (LogFC)| > 1. Genes with P < 0.01 and |log2FoldChange (LogFC)| > 2 were selected for further analysis.Heatmaps (Fig 2A) and volcano plots (Fig 2B) were generated using R (www.r-project.org).

### 2.3. Gene ontology, disease ontology, and KEGG analysis

Using The R package “ClusterProfiler” (v4.10.1) was used to analyze GO, DO, and KEGG pathways linked to DEGs.Pathways and terms with P-values below 0.05 were had significant impacts.

### 2.4. Enrichment analysis of gene sets

Gene set enrichment analysis (GSEA) was conducted using the ‘ClusterProfiler’ (v4.10.1) and ‘org.Hs.e.g.,db’ (v3.18.0) R packages, a P-value under 0.05 and a false discovery rate (FDR) below 0.5 indicate significant enrichment.

### 2.5. Selection and validation of diagnostic biomarkers

The ‘glmnet’ R package (version 4.1–8) was used to implement the LASSO regression algorithm and determine the optimal model parameters.The optimal lambda value was employed to fit the LASSO model, helping to identify diagnostic biomarkers for IA.Using the R package ‘pROC’ (version 1.18.5), we generated Receiver Operating Characteristic (ROC) curves for the identified biomarkers.

### 2.6. Immune cell infiltration analysis

Using the “IOBR” R package’s quanTIseq algorithm, scores were calculated for ten different kinds of immune-infiltrating cells.The cell types assessed included B cells, monocytes,M1 macrophages and M2 macrophages, natural killer cells, CD4 + T cells and CD8 + T cells,neutrophils,dendritic cells,and Treg cells. The correlation heatmap visualizes the interrelationships between these immune cell types.The ‘cor’ function was employed to analyze the correlation between diagnostic markers and infiltrating immune cells using the Spearman correlation coefficient.

### 2.7. Data analysis for single-cell sequencing

Seurat R package (version 5.0.3) was used to process the GSE193533 mouse single-cell sequencing dataset.Cells that more than 5,000 or fewer than 200 genes or over 20% mitochondrial gene expression, were excluded to maintain data quality.Expression values were scaled to 10,000 counts per cell to normalize the dataset for library size differences.Log normalization was then applied to the count-normalized expression matrix.Highly variable genes were identified for further analysis.Dimensionality reduction was achieved using Principal Component Analysis (PCA).A neighborhood graph was constructed from PCA data and visualized with DimPlot.

### 2.8. Analyses of statistics

Using the stats package within R,for group comparisons, independent sample t-tests were used, with a significance level of P < 0.05.

## 3. Results

### 3.1. Collecting and analyzing differentially expressed gene data

A GEO database was searched for raw expression profiles for the required datasets.The ComBat function was utilized to address batch effects from varying sequencing platforms and processing batches in datasets GSE6551, GSE15629, GSE26969, GSE54083, GSE75436, and GSE13353.The Principal Component Analysis (PCA) plot distinctly separated samples from the six datasets.Upon the removal of batch effects, the samples from the six datasets were intermixed, indicating successful elimination of batch effects.The Surrogate Variable Analysis (sva) function was employed to mitigate inter-dataset batch effects.This was corroborated by the box plot, which initially displayed inconsistencies among the samples; however, post batch effect removal, the samples exhibited uniformity, thereby further validating the effective elimination of batch effects (Fig 1B-C).After merging and normalizing the datasets, we identified 13,101 DEGs between normals and intracranial aneurysms, using a threshold of |Log2FC| > 1.Among these, in the group with intracranial aneurysms, 1,108 DEGs were upregulated, while 969 DEGs were downregulated (Fig 1D-E).

### 3.2. DEGs analysis: functional, disease, and pathway enrichment

DEGs identified in aneurysms were examined by GO, DO, and KEGG pathway enrichment analyses.An analysis of GO enrichment revealed significant enrichment of DEGs in biological processes including muscle contraction,muscle system processes,regulation of muscle contraction,cardiac conduction,and regulation of blood circulation.Cellular components (CC) significantly enriched included contractile fibers, Z lines, I bands, sarcomeres, and myofibril bundles.Significantly enriched molecular functions (MF) included scaffold protein binding, extracellular matrix structural components, cytoskeletal structural components, enzyme inhibitor activity, and muscle structural components (Fig 2A-B).

In the DO enrichment analysis, DEGs were primarily associated with myocardial infarction, hyperthyroidism, aortic aneurysm infection, and other aortic diseases.This association provides a useful reference for identifying relevant biological processes and pathways.DEGs were mainly associated with immune-related pathway enrichment analysis using KEGG, including cytoskeleton organization in muscle cells, calcium signaling, and Staphylococcus aureus infection.It appears that immune response mechanisms play a critical role in intracranial aneurysm progression and development.

**Fig 3:**
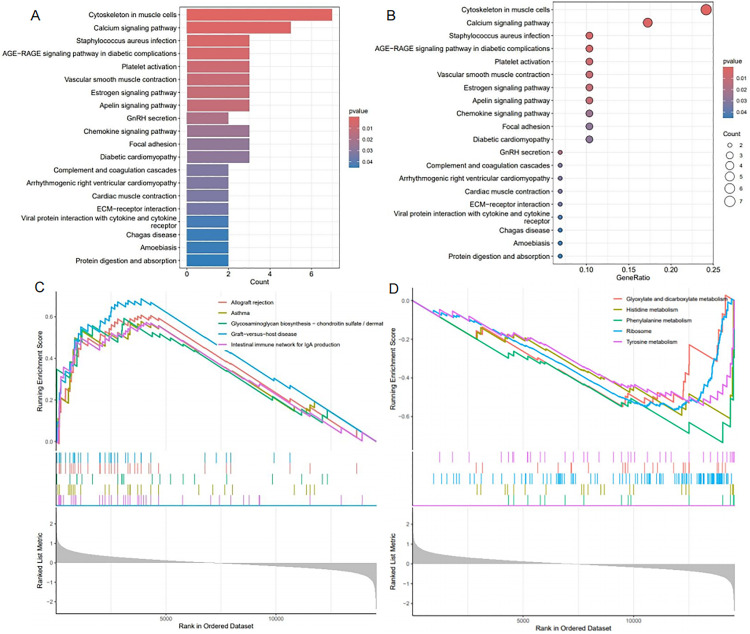
Pathway Enrichment Analysis of DEGs (A-B: Bar chart (A) and bubble chart (B) of KEGG enrichment analysis for DEGs in the datasets; C-D: GSEA enrichment plots for upregulated pathways (C) and downregulated pathways (D) for DEGs in the datasets).

Subsequent GSEA indicated a significant enrichment of the graft-versus-host disease pathway among the upregulated pathways in the intracranial aneurysm cohort (Fig 3C), whereas the phenylalanine metabolism pathway was notably enriched among the downregulated pathways in the same group (Fig 3D).

### 3.3. Identification of pathogenesis-related genes

**Fig 4:**
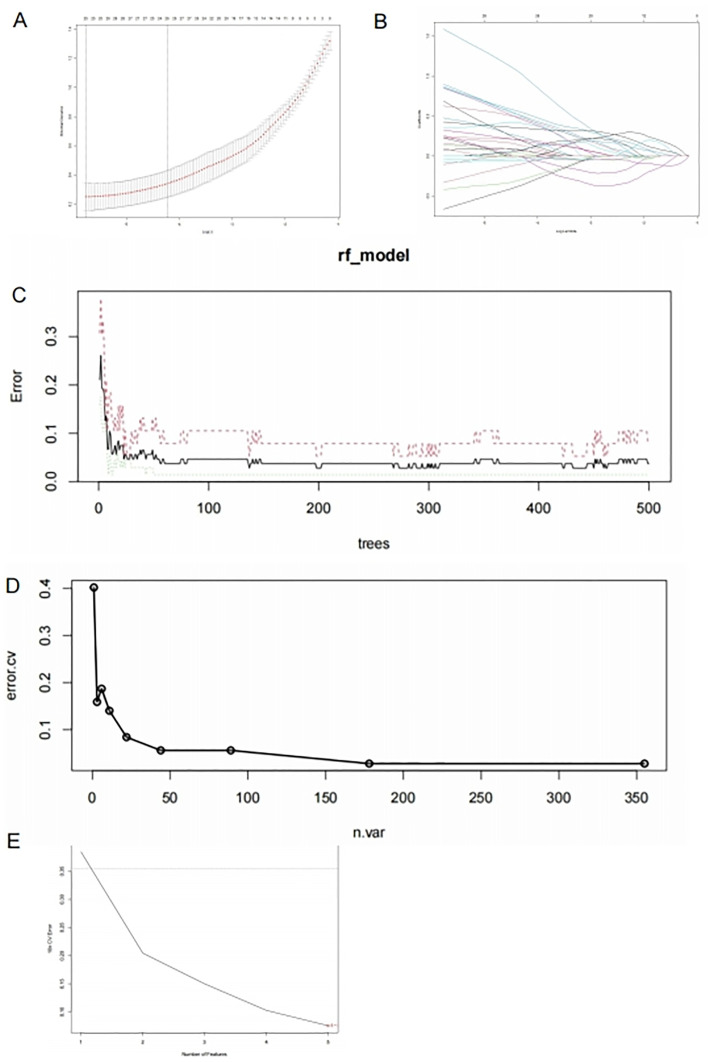
Identification of Pathogenesis-Related Genes (A: Cross-validation curve of LASSO regression, determining the optimal model position. B: Convergence plot of regression coefficients, showing the convergence of coefficients for pathogenesis-related genes selected by the LASSO model. C: Relationship between overall error rate and the number of trees in Random Forest. D: Recursive feature elimination using cross-validation in Random Forest, indicating a stable error rate at 44 features. E: Relationship between generalization error and the number of features.).

Using the LASSO regression algorithm, 25 genes were identified as significantly associated with intracranial aneurysm pathogenesis from the DEGs (Fig 4A-B). Additionally, the Random Forest algorithm identified the top 44 genes (Fig 4C-D), while the SVM-RFE determined the top 20 genes (Fig 4E).

### 3.4. Validation of potential diagnostic biomarkers

**Fig 5:**
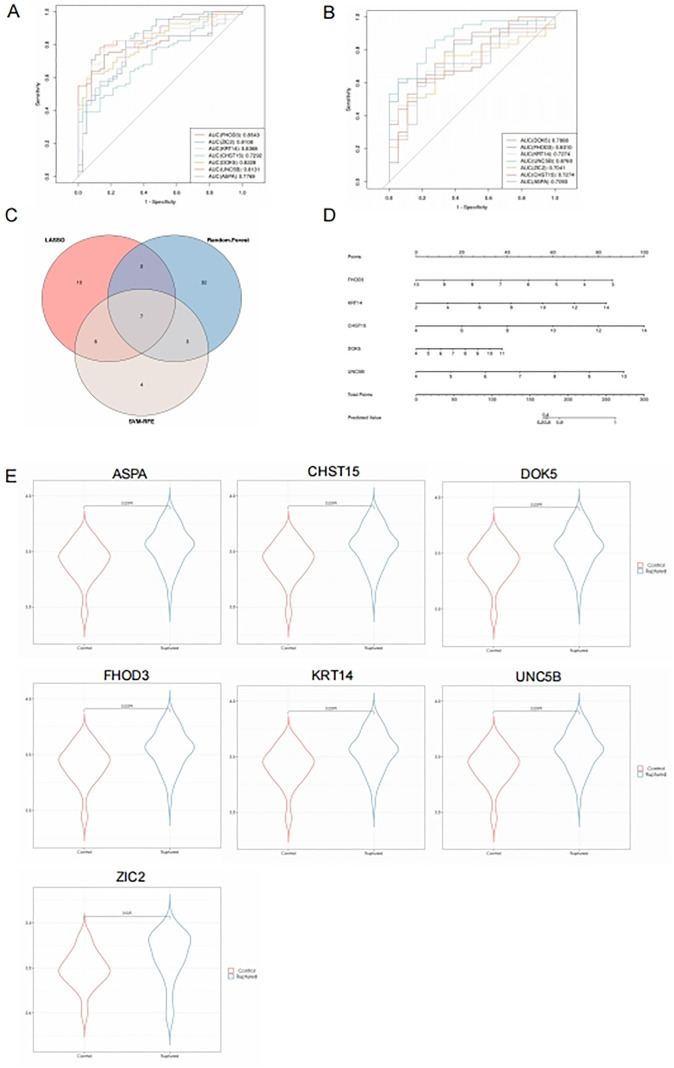
Validation of Pathogenesis-Related Genes (A: ROC curves validating the diagnostic efficacy of the seven pathogenesis-related genes. B: ROC curves for the seven genes in peripheral blood samples. C: Intersection of three machine learning methods. D: Nomogram model showing good clinical utility. E: Expression levels of the seven genes in peripheral blood samples.).

The convergence of three machine learning methodologies identified seven candidate genes: “FHOD3,” “ZIC2,” “KRT14,” “CHST15,” “DOK5,” “UNC5B,” and “ASPA” (Fig 5C). To further assess the diagnostic efficacy of these pathogenesis-related genes, In the analysis of ROC curves, all genes exhibited an AUC greater than 0.70.Notably, “UNC5B,” “DOK5,” “KRT14,” and “ZIC2” demonstrated an AUC exceeding 0.8, signifying high sensitivity and specificity. (Fig 5A).

Given the clinical challenge of obtaining aortic tissue, the diagnostic performance of these genes in peripheral blood was also evaluated. All genes maintained an AUC above 0.7, with “UNC5B” and “FHOD3” achieving an AUC greater than 0.8(Fig 5B).Predictive modeling indicated substantial clinical utility for these genes (Fig 5D), with “UNC5B” and “DOK5” showing the most pronounced differences in peripheral blood validation (Fig 5E).

### 3.5. Analyzing the amount of immune cells infiltrating

**Fig 6:**
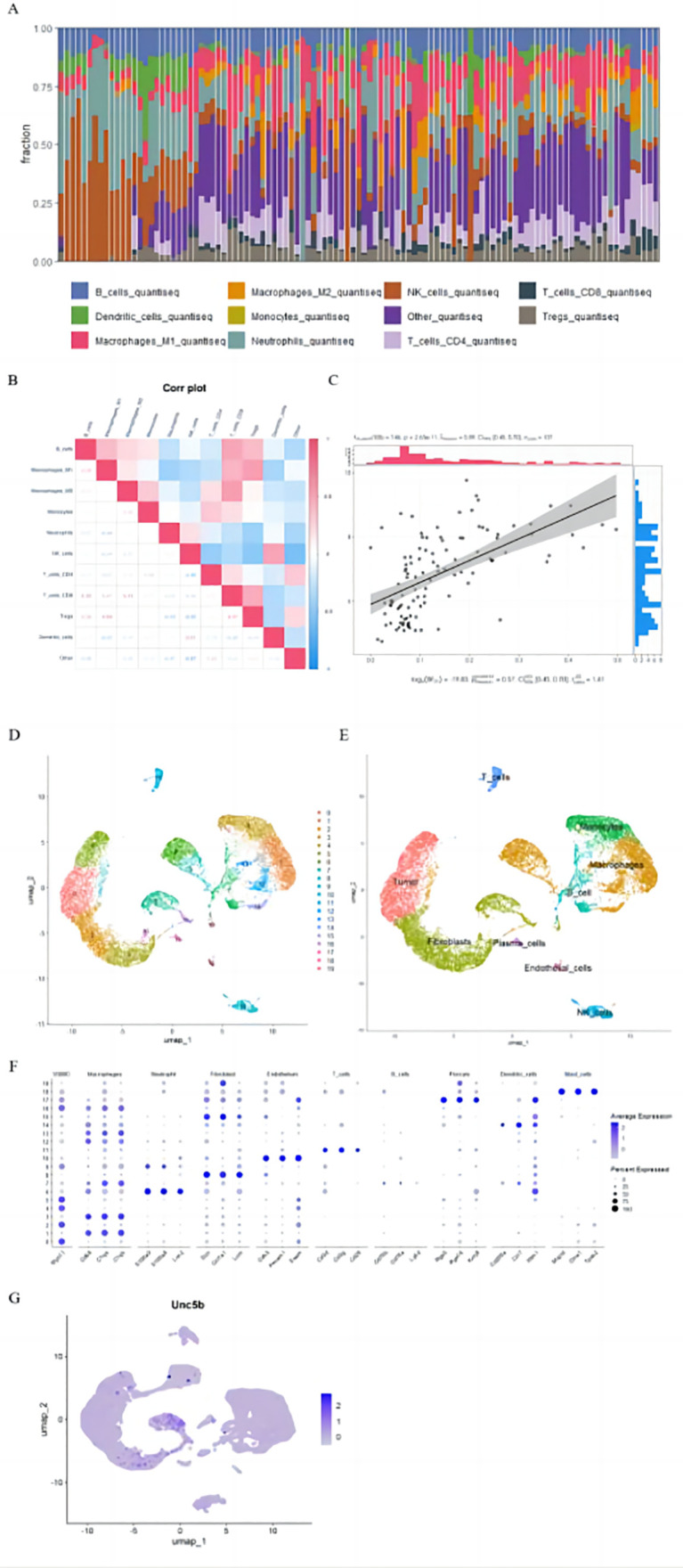
Analysis of Pathogenesis-Related Genes (A: Levels of immune cells in normal and intracranial aneurysm samples. B: Correlation heatmap of immune cells in normal and intracranial aneurysm samples. C: Scatter plot showing the positive correlation between UNC5B expression and M1 macrophage levels. D-F: Classification of single-cell subpopulations. G: Expression and distribution of the UNC5B gene.).

The quanTIseq algorithm analyzed 10 immune cell types in normal aortic and IA groups to elucidate immune responses in IA.It was determined that NK cells were significantly higher in the normal group than in the IA group, suggesting a decline in NK cells as IA progresses (Fig 6A).Dendritic cells and NK cells were positively correlated in the correlation heatmap of the ten immune cell types.Furthermore, M1 macrophages exhibited positive correlations with B cells, Tregs, and T cells.B cells showed a positive correlation with M1/M2 macrophages, monocytes, CD8 + T cells, and Tregs (Fig 6B).

To identify possible therapeutic targets for IA, correlations were examined between DOK5, UNC5B, and various immune cells.The analysis identified that ‘UNC5B’ expression correlates positively with the levels of M1 macrophages.(Fig 6C).UMAP clustering of single-cell sequencing data identified nine distinct cell types, with ‘UNC5B’ expression predominantly associated with fibroblasts (Fig 6D-F).The increased presence of M1 macrophages and elevated ‘UNC5B’ expression in fibroblasts may contribute to intracranial aneurysm pathogenesis.

## 4. Discussion

Current clinical interventions are capable of treating dilated intracranial aneurysms (IA), thereby extending patient survival.Single surgical approaches may be insufficient for treating complex aneurysms, requiring a combination of microsurgical and endovascular techniques, each with its own complications. [23]. In this study, key genes and biological processes involved in the development of intracranial aneurysms were analyzed using bioinformatics.

GO analysis revealed that DEGs are parts of biological processes such as muscle contraction, blood circulation regulation,muscle system processes, muscle contraction regulation,besises cardiac conduction.Previous research has shown significant immune cell proliferation and activation during intracranial aneurysm (IA) pathogenesis.DEGs were enriched in molecular functions including scaffold protein binding, extracellular matrix (ECM) structural components, cytoskeletal components, enzyme inhibitor activity, and muscle structural components. ECM degradation is a key pathological feature of IA, with DEGs likely contributing to vascular wall weakening and ECM destruction.This degradation renders the intracranial arterial structure more susceptible to dilation and rupture [24].

The study found that DEGs between the two groups were mainly enriched in cytoskeletal muscle cells, calcium signaling pathways, and related immune signaling pathways, according to KEGG analysis.IA research in the past has found similar results.In murine models lacking the NF-κB subunit P50,IA formation and macrophage infiltration were reduced [17]. IA formation was promoted and NF-κB expression and activation were increased by silencing the adenomatous polyposis coli (APC) gene in rats [25]. Notably, there are several risk factors for the formation and rupture of IAs, such as alcohol abuse, smoking, oxidative stress, and obesity, that make NF-κB activated and Pro-inflammatory markers are expressed more strongly and matrix metalloproteinases [26–29].

With the help of machine learning techniques, potential markers and therapeutic targets for diagnostics for IA were identified.Validation sets corroborated that “FHOD3,” “ZIC2,” “KRT14,” “CHST15,” “DOK5,” “UNC5B,” and “ASPA” are associated with IA development, with “UNC5B,” “DOK5,” “KRT14,” and “ZIC2” demonstrates potential as blood biomarkers for IA.In contrast, “KRT14” and “ZIC2” did not exhibit significant differences in peripheral blood, likely due to their primary functional roles within intracranial arterial tissues and their lack of secretion into peripheral blood.Consequently, these genes are unsuitable as circulating markers.

Early-stage IA are typically asymptomatic, the use of vascular imaging for early detection and diagnosis is common.DSA (Digital Subtraction Angiography),though invasive and time-consuming, diagnostics of IA are based on this method [30]. Increasingly, non-invasive techniques like CTA (Computed Tomography Angiography) are used.However, it has lower sensitivity for small aneurysms and requires significant radiation exposure and contrast media, when patients have renal impairments or allergies to contrast agents, this procedure may be risky [31]. MRA (Magnetic Resonance Angiography) is another noninvasive method for diagnosing IA that avoids these limitations and has similar accuracy as CTA, albeit with lower specificity [32]. Routine clinical examinations, such as blood tests, are convenient, non-invasive, cost-effective, and broadly applicable, offering timely insights into the body’s immune function and thereby facilitating the rapid and straightforward diagnosis of IA [33]. Consequently, this study proposes a clinical diagnostic strategy for IA based on these findings.

Doks, recently identified as cytoplasmic signaling molecules associated with tyrosine kinases [34], are implicated in cell differentiation and proliferation. Prior studies have demonstrated that DOK5, a member of the Doks family, neuronal cells are influenced by it in terms of differentiation and proliferation, cardiomyocytes, and tumor cells [35–37]. First reported in 2001, A mutation in DOK5 is associated with familial medullary thyroid carcinoma and multiple endocrine neoplasias. DOK5 promotes MAPK activation by c-Ret [37]. Favre et al.T cell activation regulates DOK5 expression in T cells. Pothlichet et al.The upregulation of DOK5 in human melanoma may be linked to metastasis [38,39]. This study identifies “UNC5B” and “DOK5” as potential circulating diagnostic markers for IA; however, This marker’s diagnostic efficacy needs to be further substantiated clinically.

The cell surface receptor UNC5B, specific to vascular systems, is involved in the formation and remodeling of the vascular system, as well as in axon sprouting in the nervous system [40]. In cardiovascular and neurological diseases, guidance receptors play a crucial role [41]. UNC5B expression has been notably identified in endothelial cell clusters across various tissues [42–45]. Macrophage UNC5B expression dysregulation is linked to atherosclerosis development and plaque stability [40]. Non-specific inhibition of UNC5B significantly mitigates myocardial ischemia-reperfusion injury [46],indicating its potential role in age-related diseases.While UNC5B is recognized for its role in angiogenesis within endothelial cells [47], it also functions as a critical vascular neuron.

UNC5B, a transmembrane protein of type I, has extracellular immunoglobulin-like (Ig) and type I thrombospondin (TSP) domains, ZU5 domain located in the cytoplasm (found in occludin-1 and the UNC5 family) and a death domain (DD).During vascular development, UNC5B is localized in the filopodia of endothelial tip cells, where it facilitates the repulsive response to the chemoattractant Netrin [42,48]. In endothelial cells, UNC5B acts as a dependence receptor, triggering apoptosis without its ligand Netrin-1 and inhibiting apoptosis when Netrin-1 is present, thus regulating developmental angiogenesis [46,49,50]. The molecular mechanisms behind UNC5B-mediated apoptosis are not well understood, yet they are crucial for understanding developmental and tumor angiogenesis.A proper equilibrium between pro-apoptotic and anti-apoptotic signals is essential for maintaining vascular integrity [51–53]. Although pro-angiogenic factors often inhibit apoptosis [54], formation and remodeling of capillaries are dependent on the induction of apoptosis.Notably, UNC5B inhibition has been demonstrated to reduce angiogenesis in vivo [55,56].

Single-cell sequencing data reveal fibroblasts as the main source of UNC5B.The hypothesis posits that intracranial aneurysm development involves an increase in M1 cells and upregulation of the UNC5B gene in fibroblasts.The immune cell correlation heat map generated in this study supports this hypothesis.Future research will aim to further validate these findings using patient specimens and mouse models.Additionally, the UNC5B and DOK5 genes are proposed as potential diagnostic markers for intracranial aneurysms.

## Supporting information

S1 File Scatterplot showing the relationship between B-cell activity and various markers. (PNG)

S2 File Scatterplot showing the relationship between CD4 + T cells and various markers. (PNG)

S3 File Scatterplot showing the relationship between CD8 + T cells and various markers. (PNG)

S4 File Scatterplot showing the relationship between Dendritic cells and various markers. (PNG)

S5 File Scatterplot showing the relationship between M1 macrophages and various markers. (PNG)

S6 File Scatterplot showing the relationship between M2 macrophages and various markers. (PNG)

S7 File Scatterplot showing the relationship between Monocytes and various markers. (PNG)

S8 File Scatterplot showing the relationship between Neutrophils and various markers. (PNG)

S9 File Scatterplot showing the relationship between NK cells and various markers. (PNG)

S10 File Scatterplot showing the relationship between Tregs and various markers. (PNG)

S1 Table Correlation of immune cells in normal and intracranial aneurysm samples. (XLSX)

S2 Table Levels of immune cells in normal and intracranial aneurysm samples. (XLSX)

S3 Table Immune cell levels across different sample groups. (XLSX)
