# Supplementary figures and images for: From genes to diagnosis: The impact of UNC5B and DOK5 in intracranial aneurysm detection and pathogenesis
Source: PLoS One. 2026 Mar 13;21(3):e0340496. doi: 10.1371/journal.pone.0340496 (PMC12987431; doi:10.1371/journal.pone.0340496)

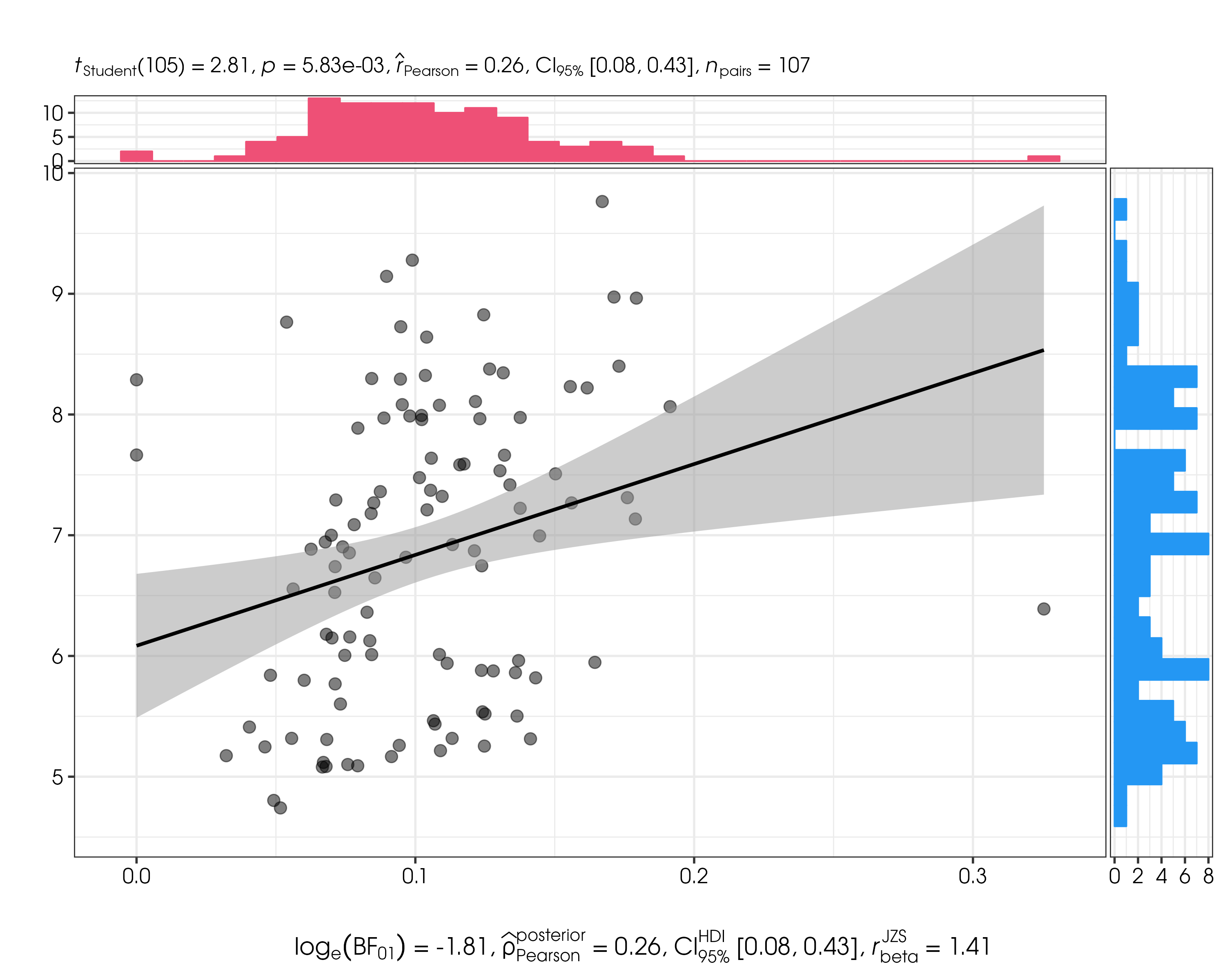

Supplement: S1 File — (PNG) [file pone.0340496.s001.png]

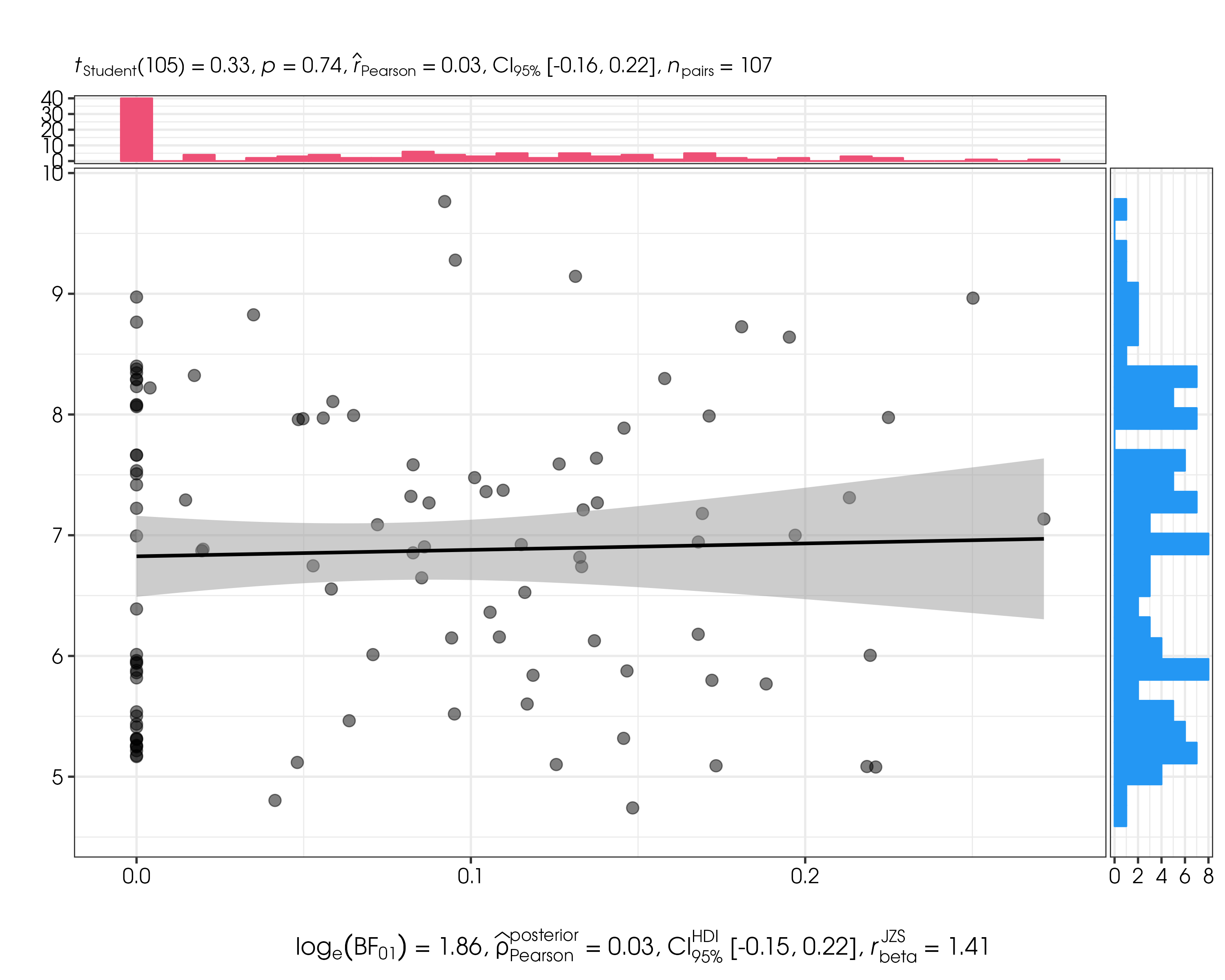

Supplement: S2 File — (PNG) [file pone.0340496.s002.png]

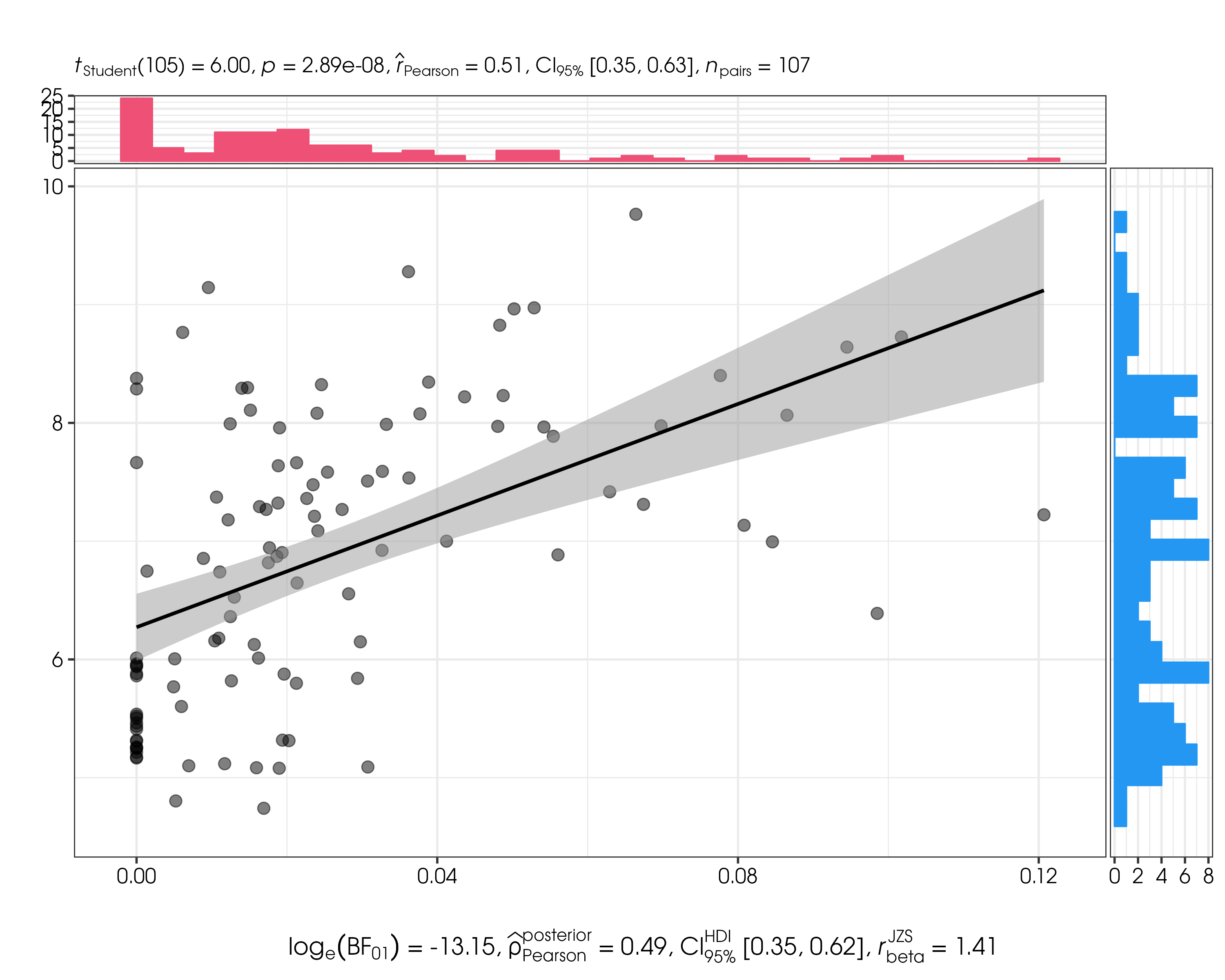

Supplement: S3 File — (PNG) [file pone.0340496.s003.png]

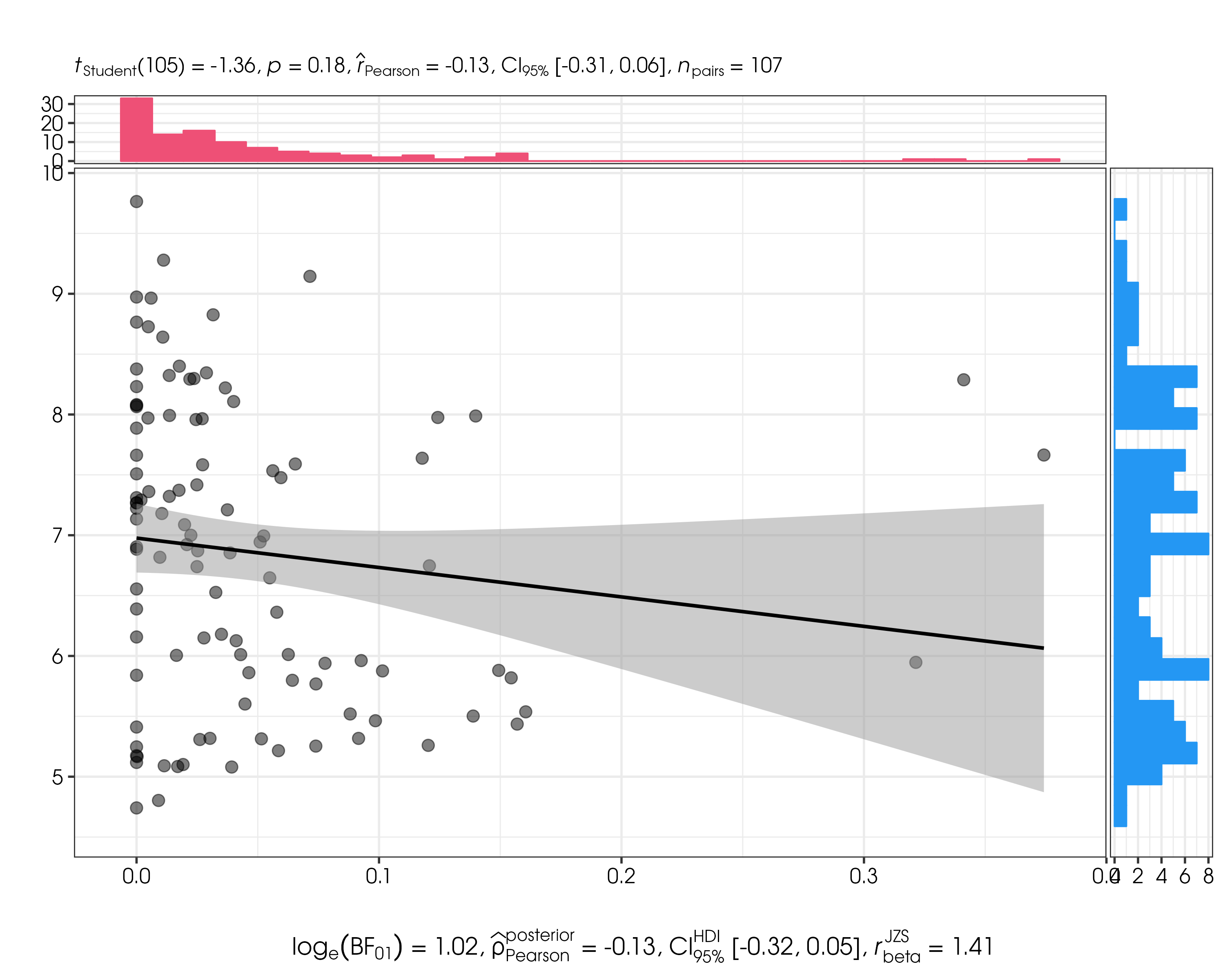

Supplement: S4 File — (PNG) [file pone.0340496.s004.png]

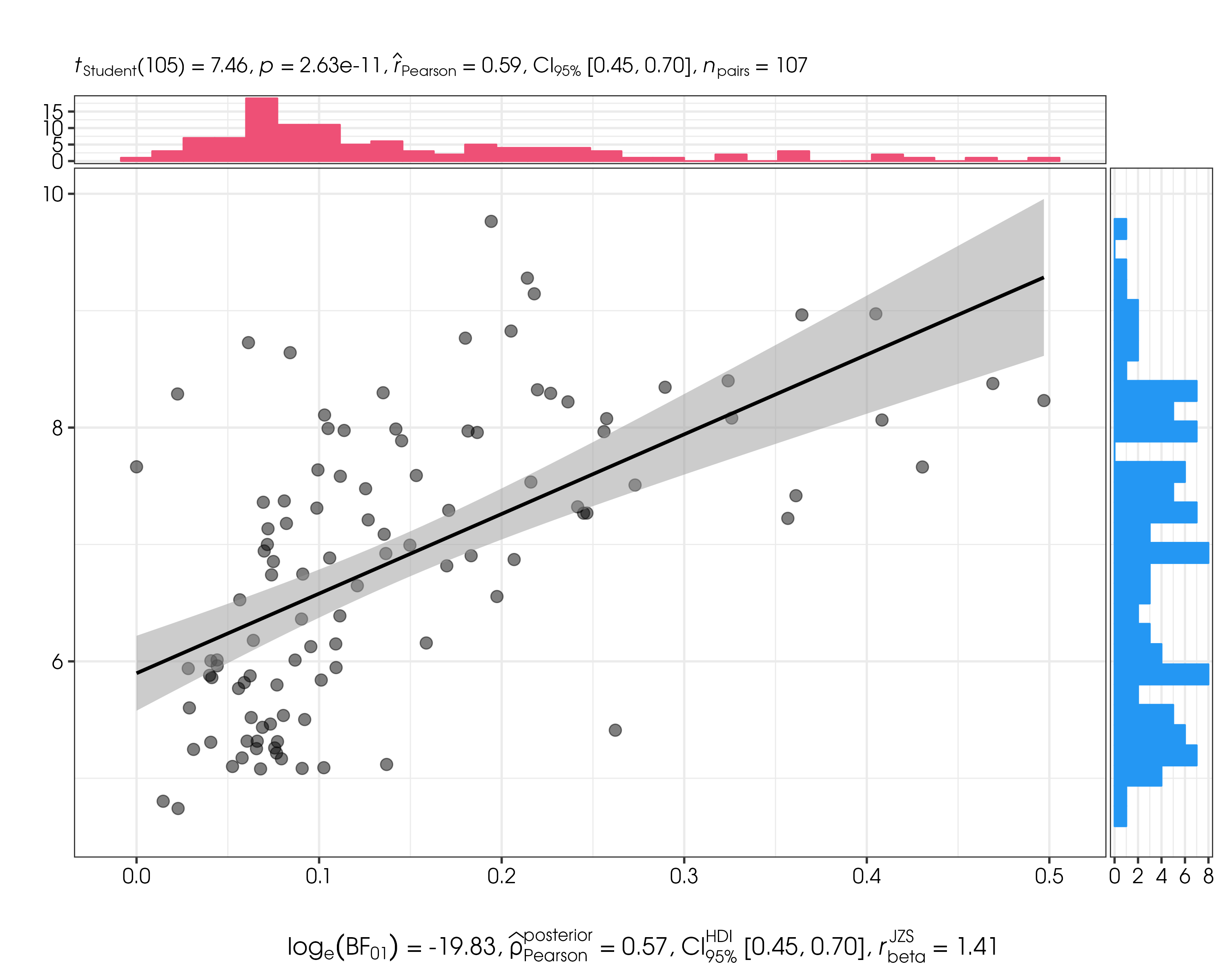

Supplement: S5 File — (PNG) [file pone.0340496.s005.png]

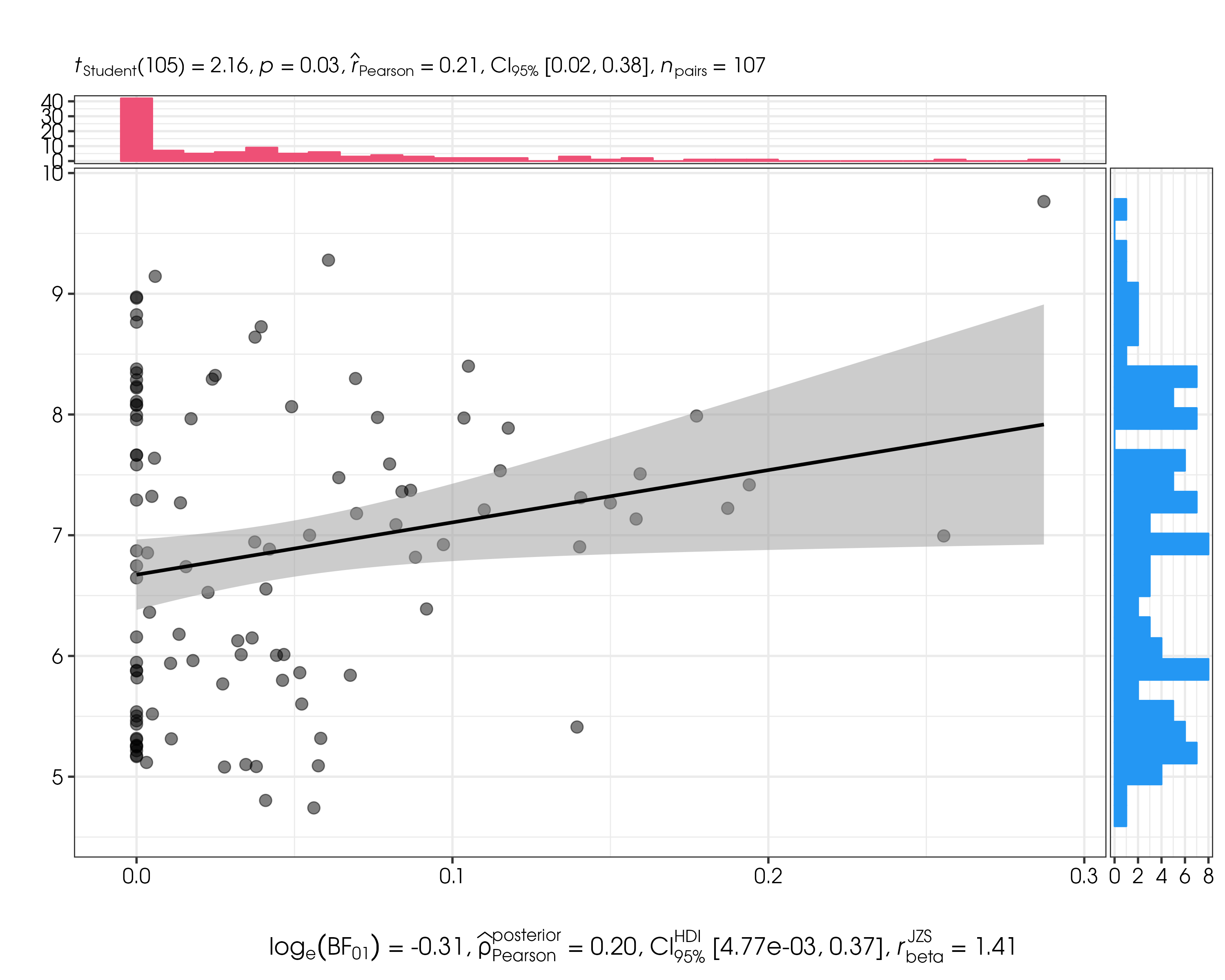

Supplement: S6 File — (PNG) [file pone.0340496.s006.png]

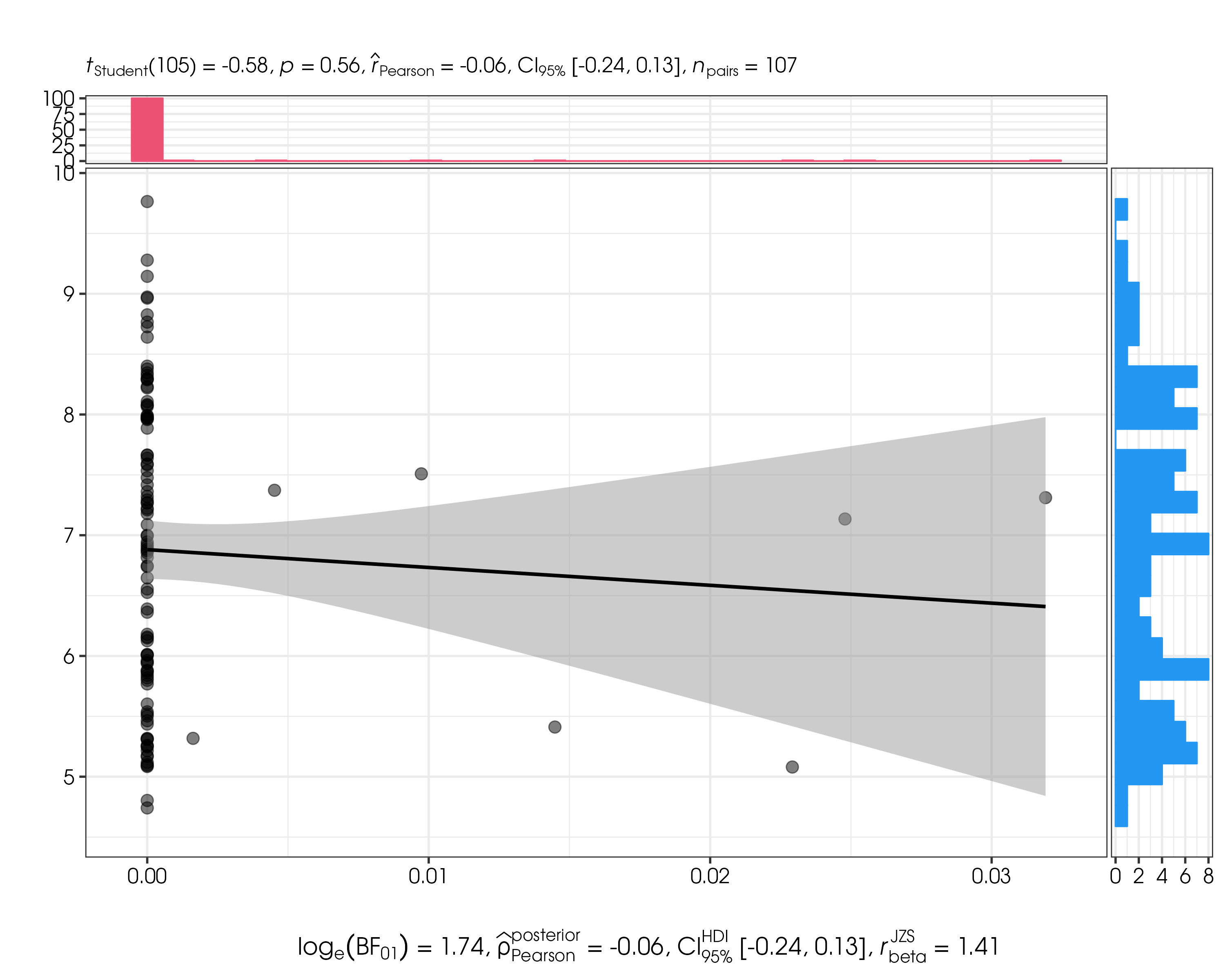

Supplement: S7 File — (PNG) [file pone.0340496.s007.png]

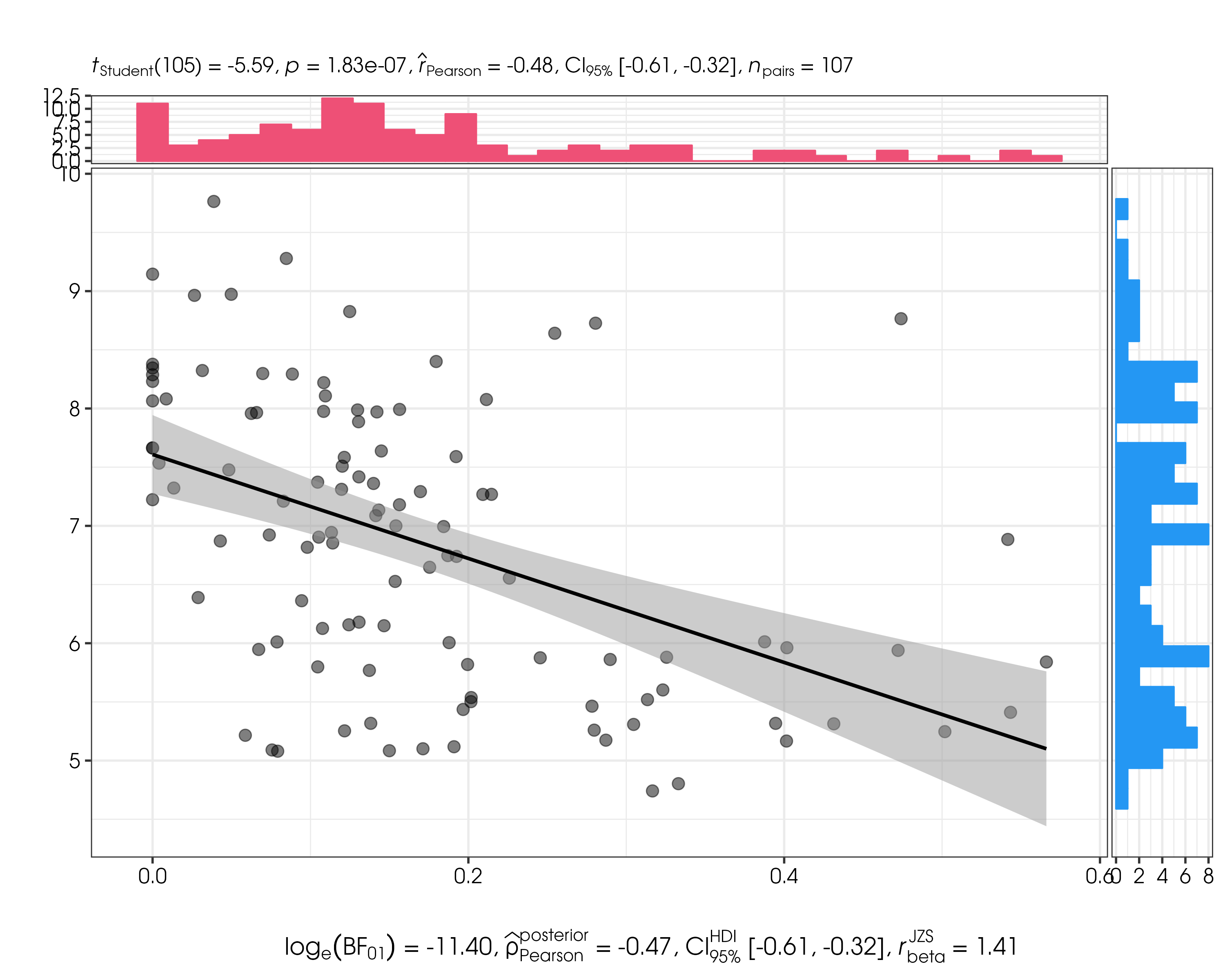

Supplement: S8 File — (PNG) [file pone.0340496.s008.png]

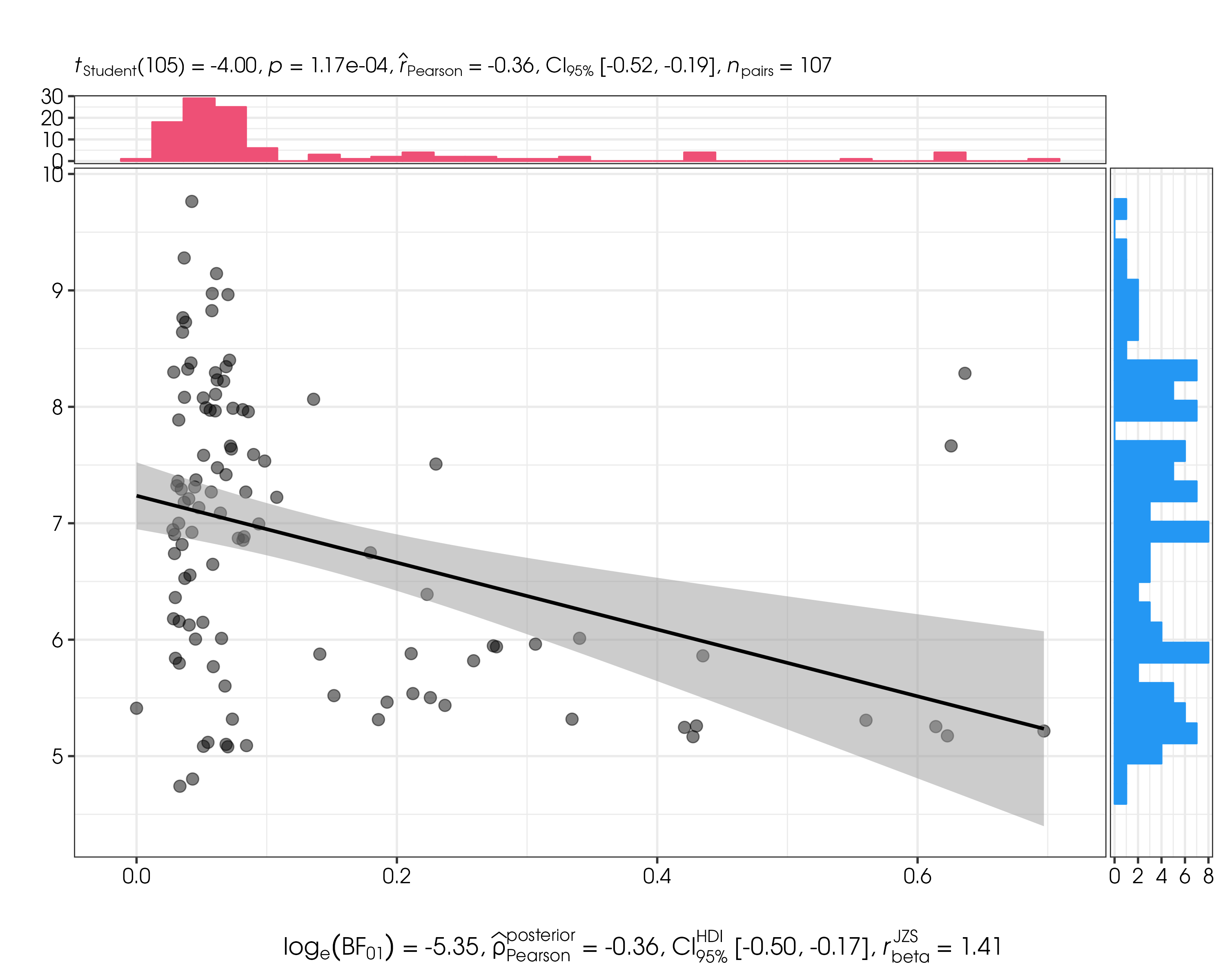

Supplement: S9 File — (PNG) [file pone.0340496.s009.png]

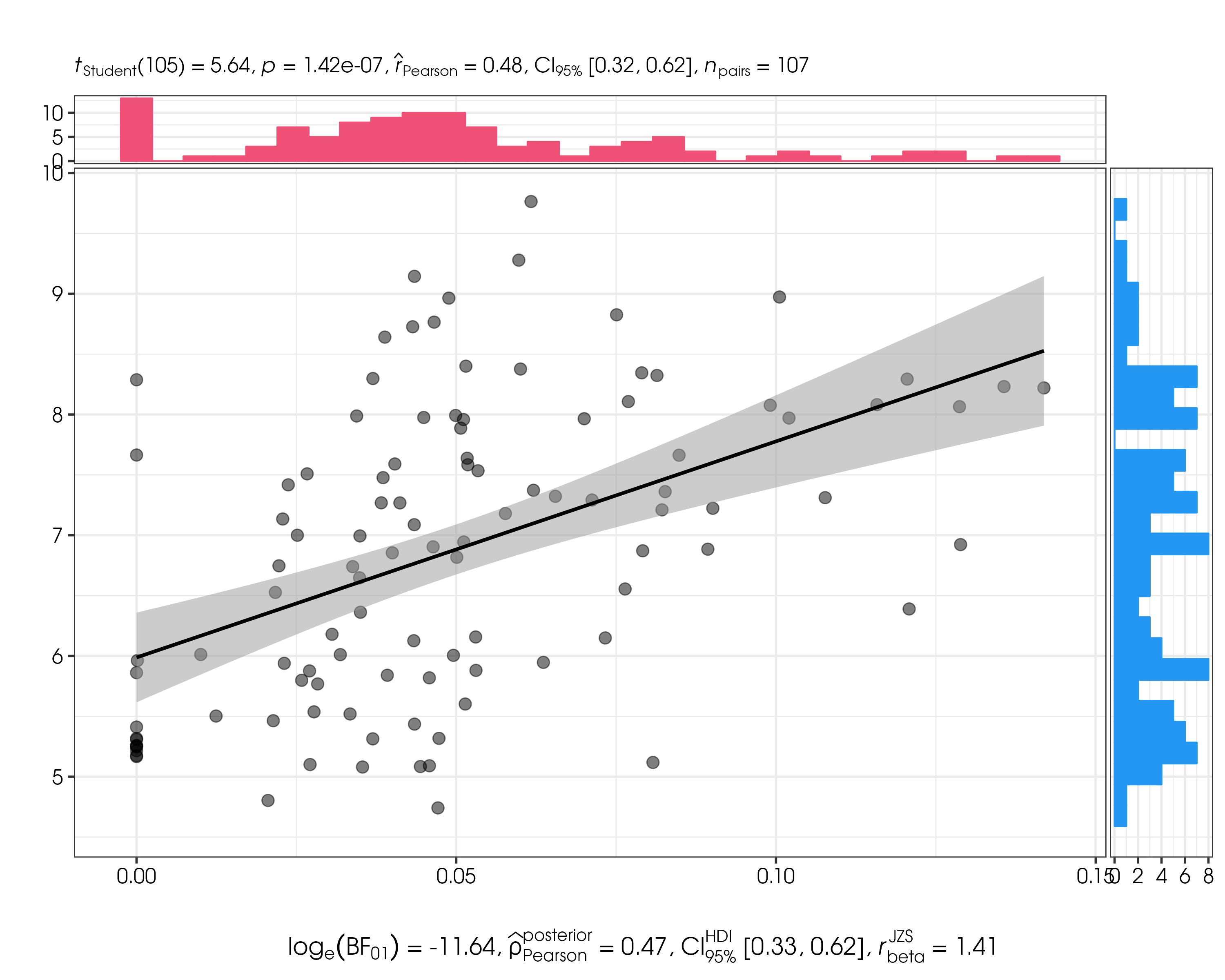

Supplement: S10 File — (PNG) [file pone.0340496.s010.png]
